# The usefulness of presepsin in the early detection of anastomotic leakage after esophagectomy

**DOI:** 10.1016/j.sopen.2025.01.003

**Published:** 2025-01-15

**Authors:** Yoshiro Imai, Ryo Tanaka, Kentaro Matsuo, Hidero Yoshimoto, Mitsuhiro Asakuma, Hideki Tomiyama, Sang-Woong Lee

**Affiliations:** Department of General and Gastroenterological Surgery, Osaka Medical and Pharmaceutical University, 2-7 Daigaku-machi, Takatsuki, Osaka 569-8686, Japan

**Keywords:** Presepsin, Esophagectomy, Anastomotic leakage, Esophageal cancer

## Abstract

**Background:**

Anastomotic leakage is a severe complication of esophagectomy, therefore early detection is crucial. Presepsin is a biomarker for early diagnosis of infectious complications. This study assessed presepsin as a biomarker for anastomotic leakage after esophagectomy, compared to C-reactive protein (CRP), white blood cells (WBCs), and neutrophils (Neuts).

**Materials and methods:**

This study enrolled 27 patients between October 2019 and December 2020. Levels of presepsin, CRP, WBCs, and Neuts were measured preoperatively and on postoperative days (PODs) 1, 3, 5, and 7.

**Results:**

Five patients had anastomotic leakage. Their presepsin levels on POD 7 were significantly higher and tended to be higher on POD 5 (*p* = 0.04 and *p* = 0.06, respectively) compared to those without leakage. The area under the curve values for presepsin were highest on PODs 5 and 7 (0.89 and 0.83). Optimal cut-off values for presepsin were 400 pg/mL (sensitivity 100 %; specificity 81.9 %) on POD 5 and similar on POD 7.

**Conclusions:**

Presepsin levels on PODs 5 and 7 effectively detect anastomotic leakage after esophagectomy, making it a valuable, simple, non-invasive early detection test.

## Highlights

• Presepsin levels on PODs 5 and 7 are effective for anastomotic leakage detection.
• Patients with leakage show markedly higher POD 7 levels.
• AUC values on PODs 5 and 7 demonstrate high accuracy.

## Introduction

Esophageal cancer is the fifth leading cause of cancer-related deaths worldwide and the seventh most common cancer worldwide [1]. Treatments for esophageal cancer are based on a treatment algorithm consisting of factors such as the tumor's clinical stage, circumference, length, and the patient's performance status [2,3]. For locally advanced esophageal cancer, esophagectomy is recommended as the primary treatment option, as long as the patient's general condition is not a concern [2,3].

Esophagectomy for esophageal cancer is one of the most invasive digestive surgical procedures. Despite the standardization of surgical techniques and perioperative management, as well as improved instrumentation, anastomotic leakage rates remain high at 13.3 %, according to the National Clinical Database (NCD) in Japan [4].

Postoperative anastomotic leakage after esophagectomy is known to cause serious complications with a risk of mortality [5,6]. Therefore, early detection of anastomotic leakage in the asymptomatic stage is crucial to preventing the development of serious complications. Reports have been made regarding the early detection of asymptomatic anastomotic leakage through computed tomography (CT), endoscopy, or contrast esophagography [[7], [8], [9], [10], [11], [12]]; however, none of these methods provide objective indicators, and thus, no established diagnostic protocol exists [13].

Presepsin, which is the soluble fraction of cluster-of-differentiation 14 (CD14), has been thought to be associated with infections [14] based on the fact that a subtype of CD14 is present inside and on the cell membranes of macrophages, monocytes, and granulocytes and is responsible for intracellular transduction of endotoxin signals. Presepsin is elevated early in the development of sepsis [15,16] and is less sensitive to invasion, such as trauma [17]. Taking advantage of these characteristics, the usefulness of presepsin in postoperative infectious complications has also been reported within the domain of gastrointestinal surgery [[18], [19], [20]]. We previously reported the usefulness of presepsin in postoperative infectious complications after gastrectomy for gastric cancer [21]. Given that esophagectomy for esophageal cancer is one of the most invasive procedures in gastrointestinal surgery, we hypothesized that presepsin, which has been shown to be less sensitive to surgical invasion, would be highly useful in detecting anastomotic leakage following esophagectomy for esophageal cancer. This study aimed to evaluate its usefulness in determining anastomotic leakage after esophagectomy for esophageal cancer compared with C-reactive protein (CRP), white blood cells (WBC), and neutrophils (Neut).

## Material and methods

### Patients

This was a single-institution prospective observational study. From October 2019 to December 2020, a total of 27 patients who underwent curative esophagectomy for esophageal cancer at the Osaka Medical and Pharmaceutical University Hospital, Japan, were included in this study. The study protocol was approved by the Ethics Committee of the Osaka Medical and Pharmaceutical University Hospital (approval no. 2020–005). Informed consent was obtained from all patients. Patients who did not provide consent for this study or who preoperatively had an active infection were excluded.

The esophageal cancer stage classification was based on the 7th edition of the tumor-node-metastasis (TNM) classification established by the Union for International Cancer Control. During the study period, patients with clinical stage 2 or 3 disease were treated with neoadjuvant chemotherapy with 5-fluorouracil and cisplatin twice every three weeks, based on the results of the Japan Clinical Oncology Group (JCOG) 9907 study [22].

### Measurements

We measured presepsin along with CRP, WBC, and Neut, which were usually measured in the perioperative period. To examine presepsin, we collected at least 2 mL of blood samples pre- and 1, 3, 5, and 7 days post-esophagectomy at 7:00 a.m. Presepsin was measured with a fully automatic immunoassay analyzer (PATHFAST, LSI Medience Corporation, Tokyo, Japan), according to the manufacturer's instructions [23]. The measurements taken after treatment administration for diagnosed anastomotic leakage were excluded from the study.

### Surgical procedures

At our institution, we perform transthoracic subtotal esophagectomy in the prone position with laparoscopic-assisted narrow gastric conduit creation and cervical anastomosis through the posterior sternal route as our standard procedure. After laparoscopic mobilization of the upper stomach, a small 7-cm incision was made in the epigastric region and transected esophagus, and the entire stomach was pulled outside the body. Using linear staplers, the stomach was divided from the lesser curvature to the fornix, resulting in a 4 cm-wide gastric conduit. Cervical anastomosis was performed with the modified Collard method using a linear stapler [24]. When the length of the gastric tube was insufficient for mechanical anastomosis, a hand-sewn anastomosis was performed. Lymph node dissection was performed based on the Esophageal Cancer Practice Guidelines 2017 edited by the Japan Esophageal Society [25]. We routinely placed a feeding jejunostomy to ensure postoperative enteral feeding and nasogastric tube through the anastomotic site into the gastric conduit.

### Perioperative management

Patients ingested 250 mL of oral carbohydrate solution in the evening before surgery and again 2 h before anesthesia. Enteral nutrition through the jejunostomy tube commenced with an initial intake of 300 kcal/day on postoperative day (POD) 1, which gradually increased to 1500 kcal/day. Fluid intake was initiated on POD 7, whereas oral intake was initiated on POD 8, confirming the absence of dysphagia. The nasogastric tube was removed on POD 2, and the removal of the cervical drain around the anastomosis site coincided with the introduction of a peroral intake. The patients were actively encouraged to ambulate on POD 1.

### Diagnosis of anastomotic leakage

Anastomotic leakage was suspected when the drainage fluid exhibited cloudiness or when signs of fever or redness around the wound were observed. To confirm the diagnosis, CT examination and contrast esophagography were conducted immediately. The presence of free air around the anastomosis or leakage of contrast medium during imaging confirmed the diagnosis of anastomotic leakage, which was defined as the date of the onset of anastomotic leakage.

### Statistical analysis

All statistical analyses were performed using JMP Pro 15 software (version 15; SAS Institute, Cary, NC, USA). Continuous variables are presented as mean ± standard deviation and were compared using the Wilcoxon rank-sum test. The chi-square test and Fisher's exact probability test were used to compare differences in categorical variables between the complication and non-complication groups. Receiver operating characteristic (ROC) analysis was performed to assess the diagnostic accuracy of infectious complications by evaluating the area under the curve (AUC). An AUC of ≥0.8 was considered to show high diagnostic accuracy, with those closest to 1 considered to be the most predictive. Statistical significance was set at *p* < 0.05.

## Results

### Patients' characteristics and morbidity

In total, 27 patients who underwent esophagectomy were enrolled in this study. Of these, 18 were men and nine were women, with a mean age of 70.9 years. The American Society of Anesthesiologists (ASA) scores, Body Mass Index (BMI), surgical dates, and pathological characteristics are shown in Table 1.

**Table 1:** Patients' characteristics.

Characteristic	*N* = 27
Age, years	70.9 ± 8.6
Sex, male/female	18/9
ASA score	
1/2/3	6/19/2
BMI (kg/m^2^)	21.4 ± 3.4
Tumor location	
Ce/Ut/Mt/Lt	4/4/7/12
pStage	
I/II/III/IV	4/11/11/1
Anastomotic technique	
Modified collard method/hand-sewn	23/4
Fields of LND (2/3)	20/7
Intraoperative bleeding (mL)	111 ± 27.8
Operative time (min)	632 ± 18.9
Infectious complications (CD ≥ 2), n (%)	12 (44.4 %)
Neoadjuvant chemotherapy, n (%)	22 (81.4 %)

ASA, American Society of Anesthesiologists; BMI, Body Mass Index; Ce, cervical esophagus; Ut, upper thoracic esophagus; Mt, middle thoracic esophagus; Lt, lower thoracic esophagus; LND, lymph node dissection; CD, Clavien–Dindo.

Data are presented as n (%) or mean ± standard deviation.

Infectious complications occurred postoperatively in 11 patients (40 %) (Table 2). Anastomotic leakage was observed in five patients (18 %). The mean number of days to clinically diagnose anastomotic leakage was 7.4 days, ranging from 6 to 9 days after esophagectomy, with no instances of diagnosis occurring earlier than 5 days. In all cases, the diagnosis was confirmed on the day anastomotic leakage was suspected based on clinical findings.

**Table 2:** Types of infectious complications.

Infectious complications	No. of patients (%)	Date of onset Mean (range)
Anastomotic leak	5 (18 %)	7.4 (6–9)
Pneumonia	5 (18 %)	10.6 [2,[10], [11], [12], [13], [14], [15], [16], [17], [18]]
Wound infection	1 (3 %)	11

Data are presented as n (%) or mean (range).

When comparing the patients' characteristics between the non-anastomotic leakage and anastomotic leakage groups, no significant differences were found in the patients' background characteristics, including age, sex, ASA score, and surgical and pathological data; only BMI showed a significant difference (Table 3).

**Table 3:** Comparison of patients' characteristics between the non-anastomotic leakage and anastomotic leakage groups.

	Non-AL (*n* = 22)	AL (*n* = 5)	*p*-Value
Age, years	69.4 ± 1.7	77.6 ± 3.6	0.05
Sex, male/female	13/9	5/0	0.07
ASA score			
1/2/3	17/4/1	0/4/1	0.1
BMI (kg/m^2^)	20.7 ± 0.6	24.3 ± 1.3	0.03
Tumor location			
Ce/Ut/Mt/Lt	4/3/7/7/1	0/1/0/3/1	0.44
pStage			
I/II/III/IV	2/10/9/1	2/1/2/0	0.2
Anastomotic technique			
Modified collard method /hand-sewn	18/4	4/1	0.92
Fields of LND (2/3)	16/6	4/1	0.5
Intraoperative bleeding (mL)	125 ± 157.9	75 ± 56.7	0.49
Operative time (min)	595 ± 274.4	579 ± 42.0	0.89
Neoadjuvant chemotherapy, n (%)	17	5	0.23

AL, Anastomotic Leakage; ASA, American Society of Anesthesiologists; BMI, Body Mass Index; Ce, cervical esophagus; Ut, upper thoracic esophagus; Mt, middle thoracic esophagus; Lt, lower thoracic esophagus; LND, lymph node dissection; CD, Clavien–Dindo.

Data are presented as n (%) or mean ± standard deviation.

### Biomarker levels

**Fig. 1:**
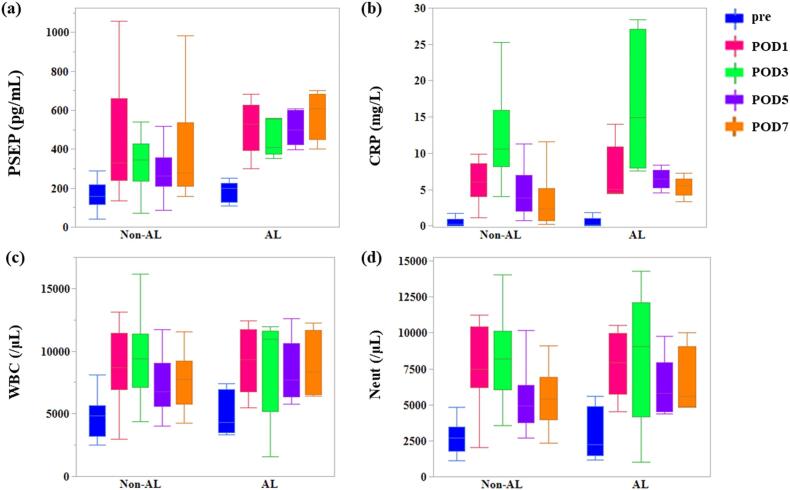
Box plot showing perioperative changes of each biomarker (a) Presepsin, (b) CRP, (c) WBC, (d) Neut. Left side, non-anastomotic leakage group; right side, anastomotic leakage group CRP, C-reactive protein; WBC, white blood cell; Neut, neutrophil; AL, anastomotic leakage. *:p < 0.05.

The box plots of each biomarker in the perioperative changes are shown in Figs. 1a–d. In 22 patients with non-anastomotic leakage, the median presepsin levels were 158 pg/mL (25th to 75th percentile: 116–218 pg/mL), 331 pg/mL (240–661 pg/mL), 345 pg/mL (234–427 pg/mL), 263 pg/mL (209–357 pg/mL), and 275 pg/mL (209–535 pg/mL) on PODs 1, 3, 5, and 7, respectively. The median presepsin levels in the five patients who developed anastomotic leakage were 200 pg/mL (25th to 75th percentile: 129–225 pg/mL), 530 pg/mL (393–626 pg/mL), 409 pg/mL (376–554 pg/mL), 500 pg/mL (423–600 pg/mL), and 608 pg/mL (450–682 pg/mL), respectively. Presepsin levels were not significantly different between the non-anastomotic and anastomotic leakage groups preoperatively or on PODs 1 and 3 (*p* = 0.97, 0.59, and 0.80, respectively). However, presepsin levels on POD 7 were significantly higher in the anastomotic leakage group than in the non-anastomotic leakage group (*p* = 0.04).

A tendency toward a significantly higher presepsin level was observed on POD 5 (*p* = 0.06), although the difference was not statistically significant. Regarding postoperative CRP, WBC, and Neuts levels, no significant difference was observed between the groups on PODs 1, 3, 5, and 7 (*p* = 0.63, 0.54, respectively). 0.17, 0.51, 0.35, and *p* = 0.85, 0.69, 0.68, 0.37, 0.23, and *p* = 0.77, 0.87, 0.89, 0.51, 0.23, respectively (Table 4).

**Table 4:** Comparison of changes in Presepsin, CRP, WBC, and Neuts levels, between the non-anastomotic leakage and anastomotic leakage groups.

	Non-AL (n = 22)	AL (n = 5)	p-Value	Non-AL (n = 22)	AL (n = 5)	p-Value
	Presepsin level (pg/mL)			CRP level (mg/dL)		
Preoperatively	158 (43–495)	200 (108–250)	0.97	0.21 (0.02–6.27)	0.18 (0.05–1.89)	0.63
POD 1	331 (136–1058)	530 (300–683)	0.68	6.03 (1.1–15.66)	5.1 (4.49–14.05)	0.54
POD 3	345 (70–1628)	409 (353–560)	0.64	10.61 (4.04–25.29)	14.9 (7.56–28.38)	0.17
POD 5	263 (86–1221)	500 (397–608)	0.06	3.90 (0.74–17.81)	6.48 (4.52–8.43)	0.51
POD 7	275 (159–982)	608 (401–700)	0.04	2.33 (0.25–12.91)	5.55 (3.33–7.26)	0.35
	WBC count (/μL)			Neuts count (/μL)		
Preoperatively	4855 (2510–22,040)	4310 (3310–7410)	0.85	2686 (1141–18,073)	2241 (1192–5580)	0.77
POD 1	8680 (2950–13,110)	9350 (5510–12,400)	0.69	7496 (2047–11,229)	7919 (4518–10,540)	0.87
POD 3	9420 (4380–16,170)	10,980 (1581–11,930)	0.68	8215 (3540–14,068)	9061 (1009–14,308)	0.89
POD 5	6780 (4050–11,730)	7700 (5780–12,580)	0.37	4926 (2685–10,156)	5806 (4399–9775)	0.51
POD 7	7780 (4250–11,530)	8320 (6420–12,230)	0.23	5392 (2363–9132)	5574 (4815–10,004)	0.23

AL, Anastomotic Leakage; POD, postoperative day; CRP, C-reactive protein; WBC, white blood cell; Neut, neutrophils.

The median presepsin levels on the day of anastomotic leakage diagnosis (or most recent day) was 605 pg/mL (401–700 pg/mL), representing a 204 % (100–362 %) increase compared to the preoperative level.

### Receiver operator characteristics analysis of the biomarker

**Fig. 2:**
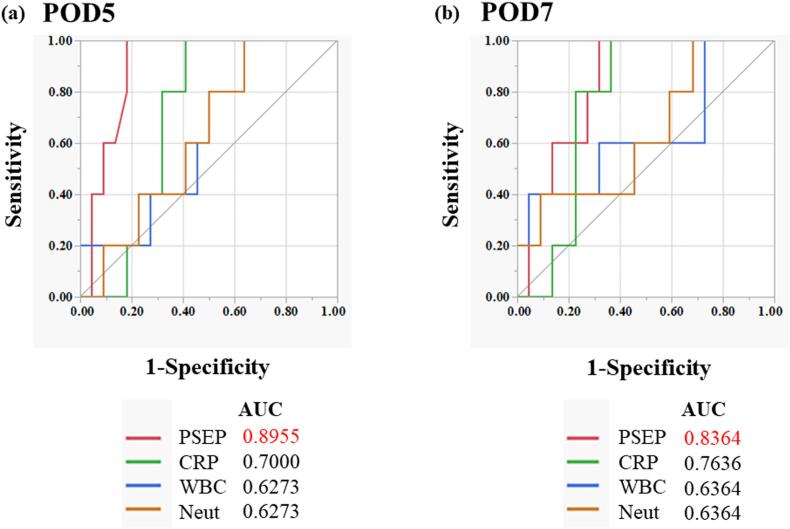
AUCs of presepsin, CRP, WBC, and Neuts according to the ROC curve (a) POD 5, (b) POD 7 ROC, receiver operating characteristic; CRP, C-reactive protein; WBC, white blood cell; Neut, neutrophil; POD, postoperative day; AUC, area under the curve.

We compared the AUC values of presepsin, CRP, WBC, and Neut on PODs 5 and 7 between the groups (Fig. 2a and b). The AUC values in the pooled prediction model were comparable to those obtained separately. The AUC values were highest for presepsin on PODs 5 and 7 (0.89 and 0.83, respectively), and these values were high (> 0.8). None of the AUC values for CRP, WBC, or Neuts exceeded 0.8.

The ROC curve was used to calculate the cut-off value for each anastomotic leakage on PODs 5 and 7 (Table 5). For presepsin, the sensitivity and negative predictive value (NPV) were 100 % on PODs 5 and 7, and the specificity was 81.9 % on PODs 5. For CRP, both the sensitivity and specificity were lower than those of presepsin at 83.3 % and 60.6 %, respectively, on POD 5. However, its specificity and positive predictive value (PPV) were higher than those of presepsin (94 % and 90 %, respectively). For WBCs and Neuts, the sensitivity and negative predictive value (NPV) were the same as those of presepsin at 100 % on POD 5; however, the specificity and PPV were extremely low at 36.4 % and 26.3 %, respectively, on POD 5. On POD 7, the specificity and PPV of WBC count were higher than those of presepsin (95.5 % and 66.6 %, respectively); however, the sensitivity was extremely low at 40 %. For Neut, the sensitivity and negative predictive value (NPV) were the same as those of presepsin at 100 %; however, the specificity and PPV were extremely low at 31.9 % and 25 %, respectively, on POD 7.

**Table 5:** Diagnostic accuracy of presepsin, CRP, WBCs, and Neuts for postoperative infectious complications on PODs 5 and 7.

	Cut-off value	Sensitivity	Specificity	PPV	NPV
POD 5					
Presepsin	397	100	81.9	55.5	100
CRP	4.52	83.3	60.6	71.4	84.6
WBCs	5780	100	36.4	26.3	100
Neuts	4399	100	36.4	26.3	100

POD 7					
Presepsin	401	100	68.2	41.6	100
CRP	4.89	75	94	90	82.3
WBCs	11,090	40	95.5	66.6	87.5
Neuts	4815	100	31.9	25	100

POD, postoperative day; CRP, C-reactive protein; WBCs, white blood cell; Neut, neutrophil; NPV, negative predictive value; PPV, positive predictive value.

## Discussion

Esophagectomy, a highly invasive surgical procedure for esophageal cancer, is associated with a notable postoperative complication rate of 41.9 % [4]. Anastomotic leakage, a specific complication, occurs at a relatively high frequency of 13.3% [4] and is associated with increased postoperative morbidity and mortality [5,6]. Therefore, the importance of preventing severe complications through early detection and prompt therapeutic intervention cannot be overstated. The study's results showed that presepsin is a useful biomarker for early determination of anastomotic leakage after esophagectomy for esophageal cancer on PODs 5 and 7.

Although contrast esophagography has been reported, its efficacy in the early detection of anastomotic leakage relies on the skill of the examiner and exhibits limited sensitivity [8,9]. Additionally, it is an invasive procedure associated with the potential risk of aspiration.

Upper gastrointestinal endoscopy has been reported as a valuable tool for early detection, allowing direct observation of the anastomosis and assessment of the mucosal surface surrounding the anastomotic site [[10], [11], [12]]. However, reports have indicated low sensitivity [13], and the examination is invasive, necessitating sedation and throat anesthesia, which also pose the risk of aspiration pneumonia. Moreover, in cervical anastomosis using the modified Collard method, observation can be challenging owing to the nonlinear configuration of the anastomotic site.

Contrastingly, Yoshiaki et al. demonstrated that asymptomatic anastomotic leakage could be identified through noninvasive means by conducting CT imaging on POD 6 [9]. Detection involved confirming the presence of air bubbles around the anastomotic site and mediastinal space. This method exhibits a sensitivity and specificity of 86.4 % and 95.8 %, respectively, and is deemed highly beneficial [9]. Nevertheless, CT examinations can be complex and contingent upon the patient's condition, as they necessitate transportation to the examination room. Some cases are difficult to distinguish because of subcutaneous emphysema and artifacts caused by the staplers and drains.

In other words, we believe that a test that can be easily conducted at the bedside is desirable. However, useful reports on the existing inflammatory biomarkers are lacking.

Presepsin measurement is a straightforward and objective test that can be conveniently conducted at the bedside. Cikot et al. reported the usefulness of presepsin in gastrointestinal anastomotic leakage [19]. However, the surgical techniques used in their study included colectomy, low anterior resection, and ileostomy closure, but not esophagectomy. Takeuchi et al. reported on the effectiveness of presepsin in postoperative infectious complications after esophagectomy for esophageal cancer [18]. While this study provides valuable insights into esophagectomy for esophageal cancer, it is essential to note that the most prevalent complication observed was wound infection, and the analysis did not include anastomotic leakage.

In this study, postoperative infectious complications generally aligned with the NCD data, with pneumonia and anastomotic leakage emerging (18 % each) as the most prevalent postoperative complications. Only presepsin levels on days 5 and 7 were elevated, with a significant difference or a tendency toward a significant difference, although not statistically significant compared to other inflammatory biomarkers. Additionally, the presepsin levels were increased before clinically suspected anastomotic leakage. The AUC value of presepsin for discriminating anastomotic leakage was highest on PODs 5 and 7 (0.89, and 0.83, respectively). Notably, both sensitivity and NPV were 100 % for PODs 5 and 7, respectively. This implies that the patient may not exhibit anastomotic leakage, potentially leading to overtreatment. However, this is advantageous for preventing serious complications. Moreover, the cutoff value remained consistently set at approximately 400 (pg/mL) for any date and time at ODs 5 and 7, enhancing its ease of interpretation. The timing of the examination for early detection of anastomotic leakage, as well as in our study, is scattered, with reports ranging between PODs 5 and 7 [[7], [8], [9], [10], [11]], and this timing is deemed appropriate because, given previous reports indicating that the onset of anastomotic leakage after esophagectomy typically occurs around POD 7 [9,11,26]. In our study, the mean day of anastomotic leakage diagnosis was on POD 7.4, which is generally consistent with previous reports. Nevertheless, given the reported early onset of POD 5.4, we considered the value of presepsin on POD 5 to be noteworthy.

This study had several limitations that warrant acknowledgment. Firstly, it is essential to note that this was a single-center study, and the findings are specific to the Japanese population. However, we believe that the anastomotic leakage rate observed in this study is comparable to that reported for NCD in Japan. Second, only presepsin and routine blood test variables, including CRP, WBC, and Neuts levels, were measured; other inflammatory biomarkers, such as procalcitonin, were not included. Therefore, comparing presepsin with procalcitonin in the future and examining whether the combined use of these inflammatory biomarkers will improve diagnostic accuracy is necessary. Third, the cohort of patients included in this study was relatively small. Hence, further investigations involving larger sample sizes across multiple institutions are needed. Finally, while the focus of this study was on anastomotic leakage, other infectious complications, such as pneumonia, may influence presepsin levels if they develop before the occurrence of anastomotic leakage. In our study, pneumonia was present in five patients (18 %), but four of them were diagnosed with aspiration pneumonia, which manifested after the initiation of oral intake. In essence, these cases occurred after the onset of anastomotic leakage and did not impact presepsin levels on PODs 5 and 7. One patient exhibited pneumonia in the acute phase with elevated presepsin levels. Because high specificity and NPV are desirable to prevent serious complications, a high presepsin level is considered acceptable if the patient develops an early infection.

## Conclusions

Presepsin levels on PODs 5 and 7 after esophagectomy for esophageal cancer are valuable biomarkers for the early detection of anastomotic leakage compared to other inflammatory biomarkers such as CRP, WBCs, and Neuts. Presepsin measurement is a highly convenient, non-invasive, and easily performed tool. Presepsin, as a biomarker, demonstrated simplicity, with constant cutoff values observed on postoperative PODs 5 and 7.

## CRediT authorship contribution statement

**Yoshiro Imai:** Writing – review & editing, Writing – original draft, Validation, Resources, Project administration, Methodology, Investigation, Formal analysis, Data curation, Conceptualization. **Ryo Tanaka:** Writing – review & editing, Investigation, Data curation. **Kentaro Matsuo:** Writing – review & editing, Investigation, Data curation. **Hidero Yoshimoto:** Writing – review & editing. **Mitsuhiro Asakuma:** Writing – review & editing. **Hideki Tomiyama:** Writing – review & editing. **Sang-Woong Lee:** Writing – review & editing, Supervision, Data curation, Conceptualization.

## Informed consent statement

The Ethics Committee of Osaka Medical College and Pharmaceutical University Hospital waived the need for informed consent because of the retrospective nature of the study.

## Ethics statement

The study protocol was approved by the Ethics Committee of the Osaka Medical and Pharmaceutical University Hospital (approval number: 2020-005).

## Funding

This research received no specific grant from any funding agency in the public, commercial, or not-for-profit sectors.

## Declaration of competing interest

None.

## Data Availability

The datasets used and/or analyzed during the current study are available from the corresponding author on reasonable request.
